# Sexual Risk Behaviors of Patients with HIV/AIDS over the Course of Antiretroviral Treatment in Northern Vietnam

**DOI:** 10.3390/ijerph15061106

**Published:** 2018-05-29

**Authors:** Thuc Minh Thi Vu, Victoria L. Boggiano, Bach Xuan Tran, Long Hoang Nguyen, Tung Thanh Tran, Carl A. Latkin, Cyrus S. H. Ho, Roger C. M. Ho

**Affiliations:** 1 Center for Research and Training, Tam Anh Hospital, 108 Hoang Nhu Tiep, Hanoi 100000, Vietnam; vuminhthuc2010@yahoo.com.vn; 2 Berkeley School of Public Health, University of California, Berkeley, CA 94720, USA; victoria.boggiano@gmail.com; 3 Institute for Preventive Medicine and Public Health, Hanoi Medical University, Hanoi 100000, Vietnam; bach.ipmph@gmail.com; 4 Bloomberg School of Public Health, Johns Hopkins University, Baltimore, MD 21205, USA; carl.latkin@jhu.edu; 5 Vietnam Young Physician Association, 64 Ba Trieu, Hanoi 100000, Vietnam; 6 School of Medicine and Pharmacy, Vietnam National University, Hanoi 100000, Vietnam; longnh.ph@gmail.com; 7 Institute for Global Health Innovations, Duy Tan University, Da Nang 550000, Vietnam; 8 Department of Psychological Medicine, National University Hospital, Singapore 119074, Singapore; su_hui_ho@nuhs.edu.sg; 9 Department of Psychological Medicine, Yong Loo Lin School of Medicine, National University of Singapore, Singapore 119228, Singapore; pcmrhcm@nus.edu.sg

**Keywords:** sexual behaviors, risk, HIV, antiretroviral treatment, Vietnam

## Abstract

Antiretroviral therapy (ART) improves the health and well-being of people living with the human immunodeficiency virus (HIV, PLWH), and reduces their risk of transmitting the virus to sexual partners. However, patterns of sexual risk behavior among HIV-positive patients taking ART in Vietnam remain largely unknown. In this study, we sought to examine sexual risk behaviors and their associated factors among HIV-positive patients receiving ART in northern Vietnam. The socio-demographic characteristics, ART use, health status, and sexual behaviors of 1133 patients taking ART in the Hanoi and Nam Dinh provinces were explored through face-to-face interviews. There were 63.5% of patients who had one sex partner, while 3.6% and 5.6% of patients had sexual intercourse with casual partners or sex workers, respectively, in the previous 12 months. Most participants tended to use condoms more often with commercial sex partners (90.2%) and intimate partners (79.7%), and less often with casual partners (60.9%). Higher age (odds ratio, OR = 1.0; 95% CIs = 1.0, 1.1) or suffering pain/discomfort (OR = 1.7; 95% CIs = 1.2, 2.4) were factors more likely to be associated with multiple sex partners. Patients who were self-employed were more likely to have sexual intercourse with casual partners/sex workers (OR = 2.1; 95% CIs = 1.1, 4.0). Meanwhile, a higher score on the EuroQol visual analog scale (EQ-VAS), an unknown HIV stage, and a longer duration of ART were adversely associated with not using condoms with casual partners/sex workers. Patients with longer durations of ART had a lower likelihood of not using a condom with casual partners/sex workers (OR = 0.5; 95% CIs = 0.3, 0.8). Our study underscored a relatively high rate of unsafe sexual behaviors among HIV-positive patients. Continuing to improve the physical and psychological well-being of HIV-positive patients in Vietnam is important in reducing the spread of HIV via risky sexual behaviors. In addition, safe-sex education should be provided to older people, and to those who are self-employed.

## 1. Introduction

Antiretroviral therapy (ART) is used to improve the lives of people living with the human immunodeficiency virus and acquired immune deficiency syndrome (HIV/AIDS, PLWH) [1,2], and to prevent the spread of the disease through both pre-exposure and post-exposure prophylaxes [3,4,5]. However, the effects of ART on sexual risk behaviors are controversial across settings. One study in rural Uganda found that risky sexual behavior decreased by 70% when HIV-positive patients received ART and counseling on healthy sexual behaviors [4]. Meanwhile, other studies indicated an increase in the engagement of patients in sexual risk behaviors after initiating ART [6,7,8]. Scheer et al. in the United States found that HIV-positive patients on highly active ART were more likely to obtain other sexually transmitted infections (STIs), which were markers of sexual promiscuity [7]. Another study found that HIV-positive men who have sex with men (MSM) on ART were more likely to engage in sexual risk behaviors, as they were less concerned about HIV transmission [9]. Thus, more data in various settings are required to develop contextualized interventions in order to reduce sexual risk behaviors among HIV-positive patients taking ART.

The HIV epidemic in Vietnam is concentrated in most-at-risk populations, namely people who inject drugs, female sex workers, and MSM. To date, there are upwards of 300,000 PLWH nationwide, and 120,000 PLWH receiving ART, with the viral suppression rate ranging from 90% to 95% [10,11,12,13]. However, while the current leading cause of HIV transmission is injection drug use [11], there was a rise in the spread of HIV via sexual risk behavior [14]. Alongside MSM and female sex workers, HIV-positive patients receiving ART who have unsuppressed viral load were also considered at-risk populations to transmit HIV via sexual behaviors [15]. There were many studies in Vietnam investigating the impact that therapies, such as methadone maintenance treatment (MMT), had on sexual risk-taking behavior, which found that MMT patients had a lower proportion of sexual risk-taking behaviors [16]. While sexual risk-taking behaviors of patients taking ART on a global scale were investigated, these patients were not studied in a Vietnamese context.

Understanding risk behaviors of patients on ART can inform the operation of HIV/AIDS programs. Currently, harm-reduction activities are targeting most-at-risk populations using mainly peer-outreach interventions, and mass media. Meanwhile, care and treatment services are facility-based, and not necessarily linked with community-based harm reduction. Therefore, preventive services are not sustained, especially for those patients on long periods of ART. In this study, we sought to examine factors associated with sexual risk-taking behaviors among HIV-positive patients taking ART in the Hanoi and Nam Dinh provinces, two HIV epicenters in northern Vietnam.

## 2. Materials and Methods

### 2.1. Study Setting

This was a sub-analysis of the 2013 HIV Services Users Survey (HSUS) dataset collected between February and July in 2013. Overall, the HSUS examined various aspects of the access, quality, and outcomes of HIV/AIDS services, from the perspectives of providers and users. Basically, a total of eight outpatient clinics located in Hanoi and Nam Dinh—2 epicenters of HIV/AIDS—were selected, including a national hospital (Hanoi), a provincial hospital (Nam Dinh), a provincial center (Nam Dinh), three district health centers from Hanoi, and two district health centers from Nam Dinh. Some articles using the same dataset and methods were published elsewhere [17,18].

### 2.2. Participants Recruitment

A convenience-sampling technique was performed to recruit participants from selected clinics. Patients were chosen if they met the following eligible criteria: (1) 18 years old or older; (2) having a HIV-positive result from the confirmatory test; and (3) agreeing to participate in the survey. Patients with cognitive impairment were excluded from the survey. Firstly, the patients were invited to a private counseling room to ensure a pleasant atmosphere, and to protect the patients’ privacy. Then, the interviewer introduced the purposes of the study, and what benefits and drawbacks patients might experience if participating in the study. We also mentioned that patients’ confidentiality would be kept, and we did not collect any data that could disclose their identity. After that, we asked patients if they would participate in the study. If the patients agreed, they were asked to give their written informed consent. A total of 1133 participants agreed to participate in the study.

### 2.3. Data Collection

Participants were interviewed face-to-face using a structured questionnaire. An interview lasted 20 min in a private room, to ensure the comfort and privacy of participants. Data collectors were master’s students in public health at the Hanoi Medical University. They were consistently trained to ensure the quality of data. 

### 2.4. Measures and Instruments

Socioeconomic information: We collected information about the general characteristics of participants, including living area (urban/rural status), age, gender, education level, marital status, and employment status.

Health status: In this study, we employed two items from the EuroQol five-dimension five-level (EQ-5D-5L) questionnaire, including (1) pain/discomfort, and (2) anxiety/depression with five levels ranging from “no problem” to “extreme problems” [19]. People who answered “no problem” were classified into the “no problem” group, while others were classified into the “having problems” group. These items were translated into Vietnamese, and validated in another study with Vietnamese HIV-positive patients [20]. Additionally, the EQ-visual analogue scale (EQ-VAS) was used to evaluate patients, which had a score ranging from 0 (the worst health state that patients could imagine) to 100 (the best health state that patients could imagine).

ART status: Data about the ART status of participants including HIV stages (asymptomatic, symptomatic, AIDS, or unknown), cluster of differentiation 4 (CD4) cell count, ART duration, whether or not they were using ART, and frequency of attending peer-to-peer meetings (meeting their peers—i.e., who were also infected with HIV—or friends in the clinics) were collected.

Sexual behaviors: We asked patients whether or not they had sex. Among those who had sex during their lifetime, we asked them to report the number of sexual partners (including intimate partners, casual partners, and sex workers) they had in the previous 12 months. We also asked whether or not they used condoms with intimate partners, casual partners, and commercial sex workers in their previous sexual intercourse.

### 2.5. Statistical Analysis

We analyzed data using STATA 12.0 (Stata Corp. LP, College Station, TX, USA). Socio-economic information (age, gender, religion, education, occupation, and marital status), health-related quality of life (HRQOL), ART status, and sexual behaviors were described. These variables were considered potential covariates in the regression models. Multivariate logistic regression was conducted to identify associated factors with sexual behaviors of patients, including having more than one partner, having sexual intercourse with casual partners/sex workers (both variables for people ever having sex during their lifetime), using a condom with spouses/intimate partners (for people having sex with intimate partners), and not using a condom with casual partners/sex workers (for people having sex with casual partners/sex workers). The results were displayed by adjusted odds ratios (aORs) and 95% confidence intervals (CIs). Stepwise strategies with forward selection were used to develop reduced multivariate models, which included variables in the models based on a *p*-value < 0.2 for the log-likelihood-ratio tests.

### 2.6. Ethics Approval 

The ethical application of this study was reviewed, and the approval was obtained by the Institutional Review Board (IRB) of the Vietnam Authority of HIV/AIDS Control’s Scientific Research Committee (28/QD-AIDS). Participation was voluntary, and patients had the right to stop the interview at any time.

## 3. Results

Table 1 highlights the characteristics of study participants. The majority were male (58.7%), lived in urban regions (77.2%), and had completed less than high school education (57.4%). Most participants (60.5%) lived with a spouse. Many were self-employed (41.4%), while others worked as workers or farmers (24.9%) or were unemployed (20.4%).

Table 2 describes that 37.7% of participants reported health problems regarding pain or discomfort, and 44.9% suffered anxiety/depression. The mean EQ-VAS score was 68.8 (SD = 17.3). Only 4.0% of patients did not initiate ART (i.e., pre-ART). The average duration of ART was 3.5 years (SD = 2.2), and the median current CD4 cell count was 294 cells/mL (interquartile range, IQR = 125–412). Most of the participants (49.5%) did not engage in peer-to-peer mentoring, while a smaller minority (24.7%) engaged in mentoring monthly.

Table 3 shows that 98.2% of patients had previously had sex, and 63.5% had one sex partner in the previous 12 months. There were 3.6% and 5.6% of patients having sexual intercourse with casual partners or sex workers, respectively, in the previous 12 months. Most participants tended to use condoms more often with commercial sex partners (90.2%) and intimate partners (79.7%), and less often with casual partners (60.9%).

Table 4 assesses factors associated with the number of sexual partners, and condom use. People having a higher age or suffering pain/discomfort were more likely to have multiple sex partners. Meanwhile, patients living with spouses/partners, and those who were white-collar workers were less likely to have more than one sex partner. Notably, patients who were self-employed were more likely to have sexual intercourse with casual partners/sex workers (OR = 2.1; 95% CIs = 1.1, 4.0) when compared with unemployed patients.

Female patients (OR = 0.3; 95% CIs = 0.2, 0.5) and people having anxiety/depression (OR = 0.6; 95% CIs = 0.4, 0.9) were less likely to use a condom with spouses/intimate partners. Meanwhile, a higher EQ-VAS score, an unknown HIV stage, and a longer duration of ART were adversely associated with not using a condom with casual partners/sex workers. Noticeably, patients with a longer duration of ART had a lower likelihood of not using condom with casual partners/sex workers (OR = 0.5; 95% CIs = 0.3, 0.8).

## 4. Discussion

The current study explored the sexual risk behaviors among HIV-positive patients taking ART. In this study, we highlighted that a minority of patients had multiple sexual partners (10%); however, the prevalence of individuals using a condom with casual partners was low. We found that HIV-positive patients’ sexual behaviors were associated with some socio-demographic and clinical factors. Our results revealed important findings about the sexual practices of this vulnerable population, and ways to create interventions that can increase safe-sex practices.

Rates of condom usage with intimate partners (79.7%) and with commercial sex workers (90.2%) mirror those that found in other countries [21], and in some cases, even surpassed those found [22]; however, rates of usage dropped to 60.9% with casual partners. The sexual practices of the patients in this study, by and large, mirrored what was found in other countries with regards to sexual risk-taking behaviors. More than 65% of the participants in this study reported no more than one sexual partner in the past year, lower than what was found in other studies in Vietnam [23], and similar to studies in Thailand and Togo [24,25].

While there were some studies suggesting that patients on ART may engage in more sexual risk-taking behaviors [7,8], ART was shown, by and large, to reduce sexual promiscuity, and transmission of HIV/AIDS among HIV-positive patients on a global scale [4,26]. In line with these global findings, in our study in northern Vietnam, we found that a longer duration of ART therapy reduced sexual risk-taking behaviors. Specifically, individuals who took ART for longer periods of time were less sexually promiscuous, signified by their reduced likelihood of having sex with casual partners/commercial sex workers without using condoms. ART duration had no impact on the number of sexual partners, or on condom use with intimate or casual partners. Overall, these findings ran alongside what was found in many other developed and developing nations with regards to ART use and sexual risk-taking behaviors [4,22,27,28], but suggested that there are opportunities for additional interventions around increasing condom use with casual partners.

Interestingly, our findings revealed that people who did not know their HIV status were less likely to have sexual intercourse with casual partners/sex workers without a condom. This suggested that perhaps those who did not know their HIV status were more worried about transmitting the virus to their sexual partners. Previous work in Uganda with patients initiating ART found that patients with lower CD4 counts were more likely to transmit HIV to their partners [29]. Patients with lower CD4 counts, who are more likely to develop AIDS, were perhaps more sexually promiscuous, as evidenced by our findings that they had a higher number of sexual partners.

Participants who were physically and psychologically impaired were more likely to engage in sexual risk-taking behaviors, as evidenced by their increased likelihood of having more than one partner, or their reduced likelihood of using condoms with intimate partners, while having a higher VAS scale leading to reduced sexual risk-taking behaviors. These findings correlated with some studies that found that depression and reduced well-being were linked to higher sexual risk-taking behaviors among patients living with HIV [30,31], but contradicted other studies that found mental state to have no impact on sexual risk taking [31,32]. Further research in Vietnam and elsewhere should explore the impact of mental state on HIV-positive and affected patients’ sexual risk-taking behaviors.

Being self-employed was a risk factor for sexual promiscuity. Self-employment was associated with an increased likelihood of having sexual intercourses with casual partners/sex workers. Perhaps individuals who are self-employed have a wider social network, which can facilitate more casual sexual events [33]. In addition, the current findings revealed that people with higher ages had a higher likelihood of having multiple sex partners, and of not using condoms with casual partners/sex workers. These results suggested that further interventions should be focused on these populations in order to reduce the risk of HIV transmission among HIV-positive patients.

Several implications can be drawn from our work. Similar to what was found among patients taking ART around the globe [4], in Vietnam, increasing the duration of ART therapy seemed to be associated with reducing sexual risk-taking behavior. Education and condom distributions should continue to target patients who are on ART, and more HIV-positive patients should be placed on ART to reduce sexual risk-taking behaviors and the spread of the virus. Patients placed on ART should be reminded that transmission of HIV is still possible despite the addition of medication, and that safe-sex practices are still important. Moreover, although patients with viral suppression have no risk of transmitting HIV to their partners, they should be reminded to adhere to the treatment in order to ensure the success of the therapy. Patients who were at later stages of HIV infection, and who reported physical and psychological issues should be reminded of safe-sex practices, and offered continuous counseling and follow-ups. Safe-sex education and condom distribution should be incorporated into treatment and care services, directed at patients who have developed AIDS. Similar levels of safe-sex education should be targeted at patients who are self-employed, as they are also more likely to have multiple sex partners. Finally, studies in other developing countries found that if patients were aware of the viral suppression effects of ART, they were more likely to engage in sexual risk-taking behaviors [29]. While we did not explore this factor in our current study, future work should investigate whether or not such knowledge acquisition is also a factor for increased sexual risk-taking behaviors among ART patients in Vietnam.

This study looked at sexual behaviors of HIV-positive patients taking ART in northern Vietnam on a large scale. However, there were a few limitations to our study. Our study methods relied on patients’ ability to recall their sexual behaviors, the duration of their ART therapy, and other elements of their health and well-being. This method of data collection is susceptible to recall bias. Our method of convenience sampling possibly led to bias, and reduced the generalizability of our results. Because we used a cross-sectional study design, we could not draw causal conclusions between ART duration and sexual behaviors; additional longitudinal data would be required for this. Several potential factors, such as duration of HIV infection, and virologic failure, were not collected, which were potential confounders affecting the results of this study. Further research is warranted to investigate the effects of these factors on the sexual behaviors among HIV-positive patients.

## 5. Conclusions

Our study underscored the importance of ART therapy in reducing sexual risk-taking behaviors of HIV-positive patients in northern Vietnam. Increasing the availability of ART, and continuing to improve the health and psychological well-being of HIV-positive patients in Vietnam are important areas of focus in reducing the spread of HIV via risky sexual behaviors. In addition, safe-sex education should be provided to older people, and to those who are self-employed.

## Figures and Tables

**Table 1:** Demographic characteristics of participants (*n* = 1133).

Characteristics	*n*	%
Gender		
• Male	665	58.7
• Female	468	41.3
Living area		
• Rural	258	22.8
• Urban	875	77.2
Education		
• <High school	650	57.4
• Highschool	362	31.9
• >High school	121	10.7
Marital status		
• Single	169	14.9
• Live with spouse	685	60.5
• Live with partner	8	0.7
• Divorced	88	7.8
• Widow	183	16.2
Employment		
• Unemployed	231	20.4
• Self-employed	469	41.4
• White collars	80	7.1
• Workers, farmers	282	24.9
• Others	71	6.3
Age (years), median (interquartile range, IQR)	34 (31–39)	

**Table 2:** Clinical characteristics and health status of participants (*n* = 1133).

Characteristics	*n*	%
Self-reported health status		
• Having pain/discomfort	427	37.7
• Having anxiety/depression	509	44.9
Human immunodeficiency virus (HIV) stage		
• Asymptomatic	456	42.1
• Symptomatic	193	17.9
• Acquired immune deficiency syndrome (AIDS)	101	9.3
• Unknown	332	30.7
Currently using antiretroviral therapy (ART)		
• Yes	1050	96.0
• No	44	4.0
Peer-to-peer meeting		
• Everyday	37	3.4
• Every week	52	4.7
• Some times per month	88	8.0
• Once per month	272	24.7
• Rarely	107	9.7
• Never	544	49.5
EuroQol visual analogue scale (EQ-VAS), mean (SD)	68.8 (17.3)	
ART duration (years), median (IQR)	4.0 (2–6)	
Current cluster of differentiation 4 (CD4) cell count (cell/mL), median (IQR)	294 (125–412)	

**Table 3:** Sexual behaviors among participants (*n* = 1133).

Characteristics	*n*	%
Ever had sex	1113	98.2
Number of sex partners in the last 12 months (*n* = 1113)		
None	21	1.9
One sex partner	707	63.5
2–3 sex partners	59	5.3
≥4 sex partners	17	1.5
Don’t remember	309	27.8
Type of sex partner in the last 12 months (*n* = 783)		
Intimate partners	755	96.4
Casual partners	28	3.6
Commercial sex partners	44	5.6
Condom use (last sexual intercourse)		
Intimate partners	596	79.7
Casual partners	14	60.9
Commercial sex partners	37	90.2

**Table 4:** Factors associated with number of sexual partners and condom use.

Factors	Having >1 Partners (Yes/No)		Having Sexual Intercourses with Casual Partners/Sex Workers (Yes/No)		Using Condom with Spouse/Intimate Partners (Yes/No)		Not Using Condom with Casual Partners/Sex Workers (Yes/No)	
	Adjusted Odds Ratio (aOR)	95% CIs	aOR	95% CIs	aOR	95% CIs	aOR	95% CIs
Age	1.0 **	1.0, 1.1					1.2 **	1.0, 1.5
Gender								
• Male	1		1		1			
• Female	1.3	0.9, 1.9	0.1 ***	0.0, 0.2	0.3 ***	0.2, 0.5		
Education								
• <High school					1		1	
• High school							10.7	0.9, 116.9
• >High school					2.6 **	1.2, 5.5	0.7	0.1, 7.2
Marital status								
• Single	1		1		1			
• Living with spouse/partner	0.1 ***	0.0, 0.1	0.2 ***	0.1, 0.4	2.2 ***	1.2, 4.0		
Occupation								
• Unemployed	1		1				1	
• Self-employed			2.1 **	1.1, 4.0				
• White-collar workers	0.4 ***	0.2, 0.8						
• Blue-collar workers/Farmers							277.6	0.8, 936.8
Living location (Urban vs. Rural)								
• Rural	1							
• Urban	1.4	0.9, 2.1			1.4	0.9, 2.3		
Having pain/discomfort								
• No	1							
• Yes	1.7 ***	1.2, 2.4						
Having anxiety/depression								
• No					1			
• Yes					0.6 **	0.4, 0.9		
EQ five-dimension (5D) VAS					1.0 *	1.0, 1.0	0.9 ***	0.8, 1.0
Current CD4 cell count					1.0	1.0, 1.0		
HIV stage								
• Asymptomatic			1		1		1	
• AIDS					0.5 *	0.3, 1.0		
• Unknown			0.5 *	0.2, 1.1			0.0 ***	0.0, 0.2
Peer-to-peer meeting								
• Daily	1		1		1		1	
• Weekly							21.0	0.4, 1024.9
• Sometimes in a month	1.5	0.8, 2.7						
• Rarely	0.5 *	0.3, 1.0	0.2	0.0, 1.7	0.6	0.3, 1.2	0.1	0.0, 1.8
• Never					0.5 ***	0.3, 0.9		
Duration of ART (years)							0.5 ***	0.3, 0.8

Note: * *p* < 0.1; ** *p* < 0.05; *** *p* < 0.01.

## References

[1] Montaner J.S.G., Lima V.D., Barrios R., Yip B., Wood E., Kerr T., Shannon K., Harrigan P.R., Hogg R.S., Daly P. (2010). Expanded haart coverage is associated with decreased population-level hiv-1-rna and annual new hiv diagnoses in british columbia, canada. Lancet.

[2] Collaborators G.H. (2016). Estimates of global, regional, and national incidence, prevalence, and mortality of hiv, 1980–2015: The global burden of disease study 2015. Lancet HIV.

[3] Attia S., Egger M., Muller M., Zwahlen M., Low N. (2009). Sexual transmission of hiv according to viral load and antiretroviral therapy: Systematic review and meta-analysis. AIDS.

[4] Bunnell R., Ekwaru J.P., Solberg P., Wamai N., Bikaako-Kajura W., Were W., Coutinho A., Liechty C., Madraa E., Rutherford G. (2006). Changes in sexual behavior and risk of hiv transmission after antiretroviral therapy and prevention interventions in rural uganda. AIDS.

[5] Porco T.C., Martin J.N., Page-Shafer K.A., Cheng A., Charlebois E., Grant R.M., Osmond D.H. (2004). Decline in hiv infectivity following the introduction of highly active antiretroviral therapy. AIDS.

[6] Almeida V.C., Donalisio M.R., Cordeiro R. (2017). Factors associated with reinfection of syphilis in reference centers for sexually transmitted infections. Rev. Saude Publica.

[7] Scheer S., Chu P.L., Klausner J.D., Katz M.H., Schwarcz S.K. (2001). Effect of highly active antiretroviral therapy on diagnoses of sexually transmitted diseases in people with aids. Lancet.

[8] Miller M., Meyer L., Boufassa F., Persoz A., Sarr A., Robain M., Spira A. (2000). Sexual behavior changes and protease inhibitor therapy. Seroco study group. AIDS.

[9] Ostrow D.E., Fox K.J., Chmiel J.S., Silvestre A., Visscher B.R., Vanable P.A., Jacobson L.P., Strathdee S.A. (2002). Attitudes towards highly active antiretroviral therapy are associated with sexual risk taking among hiv-infected and uninfected homosexual men. AIDS.

[10] Hammett T.M., Van N.T., Kling R., Binh K.T., Oanh K.T. (2010). Female sexual partners of injection drug users in vietnam: An at-risk population in urgent need of hiv prevention services. AIDS Care.

[11] Bridge J., Hunter B.M., Albers E., Cook C., Guarinieri M., Lazarus J.V., MacAllister J., McLean S., Wolfe D. (2016). The global fund to fight aids, tuberculosis and malaria’s investments in harm reduction through the rounds-based funding model (2002–2014). Int. J. Drug Policy.

[12] Rangarajan S. (2016). Factors associated with hiv viral load suppression on antiretroviral therapy in vietnam. J. Virus Erad..

[13] Tanuma J. (2017). Long-term viral suppression and immune recovery during first-line antiretroviral therapy: A study of an hiv-infected adult cohort in hanoi, vietnam. JIAS.

[14] Nguyen L.H., Tran B.X., Nguyen N.P., Phan H.T., Bui T.T., Latkin C.A. (2016). Mobilization for hiv voluntary counseling and testing services in vietnam: Clients’ risk behaviors, attitudes and willingness to pay. AIDS Behav..

[15] Huerga H., Venables E., Ben-Farhat J., van Cutsem G., Ellman T., Kenyon C. (2017). Higher risk sexual behaviour is associated with unawareness of hiv-positivity and lack of viral suppression—Implications for treatment as prevention. Sci. Rep..

[16] Boggiano V.L., Nguyen H.L.T., Nguyen L.H., Tran T.D., Van Nguyen H., Le H.T., Le H.Q., Hoang C.D., Nguyen C.T., Tran B.X. (2017). Sexual behaviors among methadone maintenance patients in a mountainous area in northern vietnam. Subst. Abuse Treat. Prev. Policy.

[17] Nguyen Q.L.T., Nguyen L.H., Tran B.X., Phan H.T.T., Le H.T., Nguyen H.D., Tran T.D., Do C.D., Nguyen C.M., Thuc V.T.M. (2017). Co-financing for viral load monitoring during the course of antiretroviral therapy among patients with hiv/aids in vietnam: A contingent valuation survey. PLoS ONE.

[18] Nguyen Q.L.T., Van Phan T., Tran B.X., Nguyen L.H., Ngo C., Phan H.T.T., Latkin C.A. (2017). Health insurance for patients with hiv/aids in vietnam: Coverage and barriers. BMC Health Serv. Res..

[19] Keebler D., Revill P., Braithwaite S., Phillips A., Blaser N., Borquez A., Cambiano V., Ciaranello A., Estill J., Gray R. (2014). Cost-effectiveness of different strategies to monitor adults on antiretroviral treatment: A combined analysis of three mathematical models. Lancet Glob. Health.

[20] Tran B.X., Ohinmaa A., Nguyen L.T. (2012). Quality of life profile and psychometric properties of the eq-5d-5l in hiv/aids patients. Health Qual. Life Outcomes.

[21] Kalichman S.C., Ntseane D., Nthomang K., Segwabe M., Phorano O., Simbayi L.C. (2007). Recent multiple sexual partners and hiv transmission risks among people living with hiv/aids in botswana. Sex. Transm. Infect..

[22] Van der Straten A., Gomez C.A., Saul J., Quan J., Padian N. (2000). Sexual risk behaviors among heterosexual hiv serodiscordant couples in the era of post-exposure prevention and viral suppressive therapy. AIDS.

[23] Thanh D.C., Hien N.T., Tuan N.A., Thang B.D., Long N.T., Fylkesnes K. (2009). Hiv risk behaviours and determinants among people living with hiv/aids in vietnam. AIDS Behav..

[24] Yaya I., Saka B., Landoh D.E., Patchali P’N M., Makawa M.S., Senanou S., Idrissou D., Lamboni B., Pitche P. (2014). Sexual risk behavior among people living with hiv and aids on antiretroviral therapy at the regional hospital of sokodé, togo. BMC Public Health.

[25] Thanawuth N., Rojpibulstit M. (2016). Sexual risk behaviors among hiv-patients receiving antiretroviral therapy in southern thailand: Roles of antiretroviral adherence and serostatus disclosure. AIDS Care.

[26] Bateganya M., Colfax G., Shafer L.A., Kityo C., Mugyenyi P., Serwadda D., Mayanja H., Bangsberg D. (2005). Antiretroviral therapy and sexual behavior: A comparative study between antiretroviral- naive and -experienced patients at an urban hiv/aids care and research center in kampala, uganda. AIDS Patient Care STDS.

[27] Crepaz N., Hart T.A., Marks G. (2004). Highly active antiretroviral therapy and sexual risk behavior: A meta-analytic review. JAMA.

[28] Dukers N.H., Goudsmit J., de Wit J.B., Prins M., Weverling G.J., Coutinho R.A. (2001). Sexual risk behaviour relates to the virological and immunological improvements during highly active antiretroviral therapy in hiv-1 infection. AIDS.

[29] Siedner M.J., Musinguzi N., Tsai A.C., Muzoora C., Kembabazi A., Weiser S.D., Bennett J., Hunt P.W., Martin J.N., Haberer J.E. (2014). Treatment as long-term prevention: Sustained reduction in hiv sexual transmission risk with use of antiretroviral therapy in rural uganda. AIDS.

[30] Parsons J.T., Halkitis P.N., Wolitski R.J., Gomez C.A., Seropositive Urban Men’s Study T. (2003). Correlates of sexual risk behaviors among hiv-positive men who have sex with men. AIDS Educ. Prev..

[31] De Vroome E.M., de Wit J.B., Stroebe W., Sandfort T.G., van Griensven G.J. (1998). Sexual behavior and depression among hiv-positive gay men. AIDS Behav..

[32] Crepaz N., Marks G. (2001). Are negative affective states associated with hiv sexual risk behaviors? A meta-analytic review. Health Psychol..

[33] Caballero-Hoyos R., Villasenor-Sierra A., Millan-Guerrero R., Trujillo-Hernandez B., Monarrez-Espino J. (2013). Sexual risk behavior and type of sexual partners in transnational indigenous migrant workers. AIDS Behav..

